# Extended-release pharmacotherapy for opioid use disorder (EXPO): protocol for an open-label randomised controlled trial of the effectiveness and cost-effectiveness of injectable buprenorphine versus sublingual tablet buprenorphine and oral liquid methadone

**DOI:** 10.1186/s13063-022-06595-0

**Published:** 2022-08-19

**Authors:** John Marsden, Mike Kelleher, Zoë Hoare, Dyfrig Hughes, Jatinder Bisla, Angela Cape, Fiona Cowden, Edward Day, Jonathan Dewhurst, Rachel Evans, Andrea Hearn, Joanna Kelly, Natalie Lowry, Martin McCusker, Caroline Murphy, Robert Murray, Tracey Myton, Sophie Quarshie, Gemma Scott, Sophie Turner, Rob Vanderwaal, April Wareham, Eilish Gilvarry, Luke Mitcheson

**Affiliations:** 1 Addictions Department, Institute of Psychiatry, Psychology & Neuroscience, Division of Academic Psychiatry, King’s College London, Addiction Sciences Building, 4 Windsor Walk, Denmark Hill, London, SE5 8AF UK; 2 South London & Maudsley NHS Foundation Trust, London, UK; 3 School of Health Sciences, Bangor University, Bangor, Wales, UK; 4 Centre for Health Economics and Medicines Evaluation, Bangor University, Bangor, Wales, UK; 5 King’s Clinical Trials Unit, Research Management and Innovation Directorate, King’s College London, London, UK; 6 NHS Tayside, Dundee, Scotland, UK; 7 Birmingham & Solihull Mental Health, NHS Foundation Trust, Birmingham, UK; 8 Addictions Division, Greater Manchester Mental Health NHS Foundation Trust, Manchester, UK; 9 Northumberland, Tyne & Wear NHS Foundation Trust, Newcastle Addictions Service, Newcastle Upon Tyne, UK; 10 Patient and Public Involvement Representative, Lambeth Service User Council, South London & Maudsley NHS Foundation Trust, London, UK; 11 Patient and Public Involvement Representative, London, UK

**Keywords:** Opioid use disorder, Long-acting injectable buprenorphine, Extended-release buprenorphine, Psychosocial intervention, Randomised controlled trial

## Abstract

**Background:**

Sublingual tablet buprenorphine (BUP-SL) and oral liquid methadone (MET) are the daily, standard-of-care (SOC) opioid agonist treatment medications for opioid use disorder (OUD). A sizable proportion of the OUD treatment population is not exposed to sufficient treatment to attain the desired clinical benefit. Two promising therapeutic technologies address this deficit: long-acting injectable buprenorphine and personalised psychosocial interventions (PSI). This study will determine (A) the effectiveness and cost-effectiveness — monthly injectable, extended-release (BUP-XR) in a head-to-head comparison with BUP-SL and MET, and (B) the effectiveness of BUP-XR with adjunctive PSI versus BUP-SL and MET with PSI. Safety, retention, craving, substance use, quality-adjusted life years, social functioning, and subjective recovery from OUD will be also evaluated.

**Methods:**

This is a pragmatic, multi-centre, open-label, parallel-group, superiority RCT, with a qualitative (mixed-methods) evaluation. The study population is adults. The setting is five National Health Service community treatment centres in England and Scotland. At each centre, participants will be randomly allocated (1:1) to BUP-XR or SOC. At the London study co-ordinating centre, there will also be allocation of participants to BUP-XR with PSI or SOC with PSI. With 24 weeks of study treatment, the primary outcome is days of abstinence from non-medical opioids during study weeks 2–24 combined with up to 12 urine drug screen tests for opioids. For 90% power (alpha, 5%; 15% inflation for attrition), 304 participants are needed for the BUP-XR versus SOC comparison. With the same planning parameters, 300 participants are needed for the BUP-XR and PSI versus SOC and PSI comparison. Statistical and health economic analysis plans will be published before data-lock on the Open Science Framework. Findings will be reported in accordance with the Consolidated Standards of Reporting Trials and Consolidated Health Economic Evaluation Reporting Standards.

**Discussion:**

This pragmatic randomised controlled trial is the first evaluation of injectable BUP-XR versus the SOC medications BUP-SL and MET, with personalised PSI. If there is evidence for the superiority of BUP-XR over SOC medication, study findings will have substantial implications for OUD clinical practice and treatment policy in the UK and elsewhere.

**Trial registration:**

EU Clinical Trials register 2018-004460-63.

**Supplementary Information:**

The online version contains supplementary material available at 10.1186/s13063-022-06595-0.

## Introduction

Opioid use disorder (OUD; DSM-5 [1]) is a debilitating and persistent addiction characterised by compulsive drug taking despite significant physical, psychological, and social harms. OUD has a high global burden of disability and mortality [2] and substantial associated social costs [3]. Many countries face an extended public health epidemic, reflected in a two-decade increase in the prevalence of fatal opioid poisoning associated with the use of heroin and non-medical pharmaceutical opioids [4]. In England and Wales, there were 4561 drug poisoning deaths in 2020 (79.5 deaths per million) with North-East England experiencing the highest rate (104.6 deaths per million) [5]. In Scotland, there were 1339 drug-related deaths in 2020, with Dundee City experiencing the highest age-standardised rate during 2016–2020 (43.1 per 100,000) and the greatest increase since 2000–2004 (5.9 per 100,000) [6].

In England in 2020, 71,034 people were enrolled in OUD treatment (almost all reporting addiction to heroin). A further 69,565 people were in treatment for OUD and co-occurring cocaine use disorder (CUD; mostly due to the smokable/base form known as *crack*). Patients with dual OUD-CUD find it harder to engage and derive clinical benefit from treatment [7]. Anxiety and depressive disorders are prevalent in the clinical OUD and CUD populations, and these moderate treatment adherence and response [8]. The nature of patients’ family and social relationships can either support or hinder treatment and recovery [9].

A daily dose of sublingual (tablet) buprenorphine hydrochloride (BUP-SL) or oral (liquid) methadone hydrochloride (MET) is the first-line, standard-of-care (SOC) maintenance pharmacotherapy for OUD. BUP is an opioid partial agonist/antagonist with actions predominantly at the endogenous *μ*-opioid and *k*-opioid receptors. MET is a full opioid agonist with actions predominantly at the endogenous *μ*-opioid receptor. In the United Kingdom (UK), there are two other BUP medications licensed for OUD: buprenorphine-naloxone (buprenorphine hydrochloride-naloxone dihydrate; Suboxone®; sublingual tablet; BUP-NX) and buprenorphine-lyophilisate (Espranor ®; sublingual wafer; BUP-ESP). BUP-NX contains the opioid antagonist naloxone (1:4 ratio with BUP) as a deterrent to injection of non-medical opioids.

In the UK, SOC medications for OUD are prescribed by primary and secondary care services and dispensed — initially through directly observed dosing — at community retail pharmacies. Community National Health Service (NHS) treatment is delivered by a multi-disciplinary team including psychiatry, nursing, psychology, and social work specialties. Patients are offered medical management for the physiological aspects of OUD and are supported to directly or indirectly receive treatments for medical conditions and adjunctive psychosocial interventions (PSI). At admission, patients are assigned to a member of the team (known as a keyworker) for case co-ordination. After an initial period of adherent medication maintenance, patients can receive progressively increasing take-home doses for self-administration, to a typical maximum of 14 days for a single dispensing event. If the patient can adhere to maintenance medication treatment, they are expected to achieve suppression in opioid use and improvements in their health status and social functioning [10–13]. Treatment retention is associated with a substantial reduction in the risk of unintentional fatal opioid poisoning (overdose) [14–18] blood-borne viral infections [19], and crime [20].

There is mixed evidence from randomised controlled trials (RCTs) on the relative effectiveness of BUP-SL and MET maintenance treatment. Flexible higher-dose MET appears to be associated with greater retention and suppression of heroin use [11, 12]; although among patients with pharmaceutical opioid dependence, there appears to be no evidence that one medication is superior [21].

There are three areas of concern about BUP-SL and MET. Firstly, a proportion of the patient population does not reduce or abstain from drug taking during maintenance treatment [22, 23]. For example, in an English national study of 12,745 patients who were enrolled in BUP-SL or MET for 12–26 weeks, 64% were using heroin on 10 or more days in past month, while 3% had deteriorated to more frequent opioid use than at admission [24]. A subsequent national cohort study observed that only 22% of patients were abstinent from heroin and cocaine when they left maintenance treatment, with crack use at admission predicting reduced likelihood of completing treatment successfully (adjusted odds ratio 0.90; 95% confidence interval 0.85–0.95) [25].

Secondly, despite the collaborative effort of prescribers and patients to select a medication and optimise the dose for suppression of opioid use, many patients leave treatment prematurely. A 1-year retention rate for SOC maintenance medication has been reported to be 57% [26]. Patients leave treatment early for several reasons. One study reported that some feel that directly observed dosing is stigmatising, and this motivates their decision to discontinue [27].

Thirdly, while retention is a key clinical objective so that patients are sufficiently exposed to medication and other services and interventions, some stay in treatment, but struggle to reduce their use of illicit/non-medical opioids. For example, among an English national OUD treatment cohort retained continuously over 5 years in SOC, 15% used heroin at a level that was essentially unchanged from admission [28].

Taken together, the research literature highlights a need to increase the effectiveness of treatment for OUD. One avenue to address this goal has been to develop better BUP delivery. Using the polymer ATRIGEL® delivery system, Indivior developed a subcutaneously injected, extended-release formulation of BUP (RBP6000). RBP6000 releases BUP for a minimum of 28 days, thereby facilitating monthly maintenance dosing. Development studies in the United States of America (USA) reported that RB6000 releases a relatively high and stable dose of BUP, achieving durable blockade of the subjective effects of opioids among people with moderate–severe OUD [29]. A subsequent double-blind randomised controlled trial (RCT) reported substantially higher abstinence for RBP6000 when contrasting 100 mg and 300 mg for maintenance versus placebo [30]. RBP6000 is now licensed as Sublocade® in the USA (BUP-XR herein for this study).

Therefore, there is potential for BUP-XR to be superior to SOC medication through (1) delivery of therapeutic levels of medication that can attenuate craving and block the subjective effects of non-medical opioid use, (2) prevention or minimisation of breakthrough opioid withdrawal symptoms, (3) flexible monthly dosing for patients that struggle to schedule their time, and (4) offer or an alternative to observed daily SOC dosing. However, there has been no head-to-head comparison with SOC medications. This is now the crucial comparison for clinical practice and policy.

A parallel approach to achieving better treatment effectiveness lies with adjunctive PSI. There has been relatively modest success from standardise (therapy manual-driven) approaches [31]. OUD is a complex phenotype, so an idiographic, personalised approach might be more fruitful [32, 33]. Support for a case formulation and pluralistic using a toolkit of interventions has been secured from a recent RCT among patients retained but treatment-resistant to BUP-SL or MET [34]. To date, there have been no published studies of BUP-XR and personalised PSI.

Accordingly, the *Ex*tended-release *P*harmacotherapy for *O*UD (EXPO) study will evaluate the effectiveness of BUP-XR versus comparison to BUP-SL and MET. EXPO will also evaluate the effectiveness of BUP-XR with adjunctive PSI in comparison with SOC and PSI.

## Methods

### Design

**Fig. 1:**
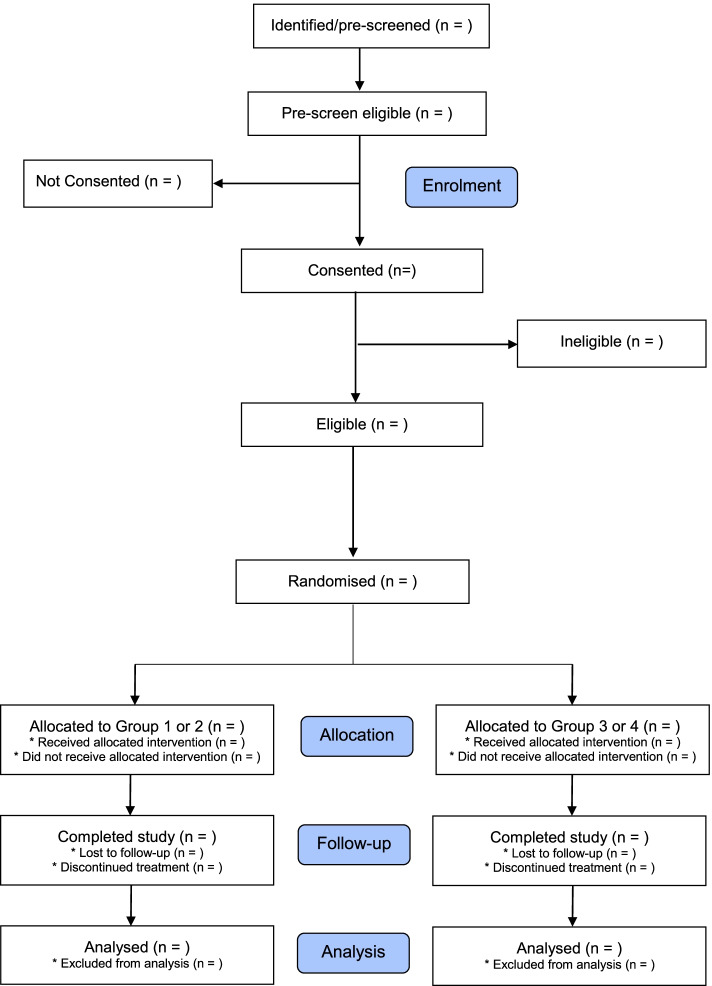
CONSORT flow of participants

EXPO is a pragmatic, multi-centre, open-label, four-arm, parallel-group, superiority RCT, with a qualitative (mixed-methods) evaluation. The co-primary aim of the study is to determine the effectiveness and cost-effectiveness of BUP-XR versus BUP-SL or MET. There will be 24 weeks of study treatment for the endpoint evaluation (Fig. 1). Participants allocated to BUP-XR can request to receive longer-term maintenance treatment for the duration of the study. EXPO also contains a single-site evaluation of the effectiveness of BUP-XR and personalised PSI versus SOC and personalised PSI. After completing the study, each participant’s treatment episode will continue following local practice (Fig. 1).

The conduct of EXPO will follow the principles of the Declaration of Helsinki [35], the Medical Research Council Guidelines for Good Clinical Practice [36], and the NHS Research Governance Framework [37]. This protocol has been written following the SPIRIT checklist for intervention trials [38] (Table S1 in supplementary material).

### Population and setting

The study population is adults (≥18 years), enrolled in SOC BUP-SL or MET treatment for OUD. The setting is community addiction treatment operated by the NHS in England and Scotland. There will be five participant recruiting centres: South-East England (Brixton, South London; study co-ordinating), North-East England (Newcastle), West-Midlands England (Solihull and Wolverhampton), North-West England (Manchester), and Tayside, Scotland (Dundee).

### Groups

At each study centre, participants will be randomly allocated to one of two groups for 24 weeks of treatment:

- Group 1: long-acting injectable BUP (BUP-XR; the experimental condition)
- Group 2: SOC medication (BUP-SL or MET; the control condition)

At the EXPO co-ordinating centre in South London, there will also be random allocation of participants to two additional groups:

- Group 3: long-acting injectable BUP with adjunctive PSI (BUP-XR with PSI; the experimental condition)
- Group 4: SOC medication with adjunctive PSI (BUP-SL or MET with PSI; the control condition)

### Primary aims

The primary aim of the EXPO study is to determine the:

1. Effectiveness and cost-effectiveness of BUP-XR versus BUP-SL or MET
2. Effectiveness of BUP-XR with PSI versus BUP-SL or MET with PSI

### Secondary aims

Secondary study aims will determine the:

1. Safety of BUP-XR
2. Retention of BUP-XR and SOC; BUP-XR with SOC and SOC with PSI
3. Effectiveness of BUP-XR and SOC to reduce opioid craving
4. Effectiveness of BUP-XR and SOC and BUP-XR with PSI and SOC with PSI to reduce use of heroin, cocaine, and benzodiazepines
5. Effectiveness of BUP-XR and SOC and BUP-XR with PSI and SOC with PSI to improve social functioning and recovery
6. Cost-effectiveness of BUP-XR versus SOC, based on incremental cost per quality-adjusted life years (QALYs) gained

These primary and secondary aims will be evaluated using pre-registered statistical and health economic analysis plans.

### Informed consent

Informed consent will be obtained from all study participants before screening by the chief investigator (CI), principal investigator (CI), or sub-investigator (SI). At each centre, participants will be enrolled in BUP-SL or MET maintenance treatment. There will be a minimum of 24 h from the point that a participant receives their Participant Information Sheet (PIS) to randomisation.

### Participant eligibility criteria

The following patient inclusion and exclusion criteria will be assessed by a medically qualified study investigator.

#### Eligibility criteria

Patients will be eligible to take part if they meet the following inclusion criteria:

1. Aged ≥ 18 years (no upper age limit; typically, patients are 25–60 years of age)
2. Current diagnosis of DSM-5 OUD via SCID-5-RV (moderate–severe at baseline for current episode)
3. Currently enrolled on MET (30 mg/day or less) or BUP-SL or BUP-NX (24 mg/day or less) or BUP-ESP (18 mg/day or less) and in the view of the clinician would be able to convert to BUP-XR within 7 days post-randomisation
4. Voluntarily seeking treatment and able to attend the clinic as required in the protocol
5. Able to communicate in English to level required to accept standard care and psychosocial intervention
6. Possession of a contactable personal mobile phone or landline telephone number and ability to nominate at least one locator individual with a verifiable address and a telephone number to assist with the arrangement of follow-up appointments
7. Living circumstances judged to be of sufficient stability to be able to engage/adhere to the study protocol
8. Is not pregnant (confirmed) or breast feeding and, if currently or intending to have potentially procreative intercourse, agrees to use a birth control method (either oral hormonal contraceptives, barrier [condom or diaphragm], or Nexplanon implant) for the duration of the study

#### Exclusion criteria

Otherwise, eligible patients will not be able to join the study if one or more of the following exclusion criteria are met:

1. Clinically significant medical condition or observed abnormalities on physical examination or laboratory investigation, including but not limited to:
  - Uncontrolled hypertension; significant heart disease (including angina and myocardial infarction in past 12 months); any cardiovascular abnormality which, in the investigator’s judgement, is clinically significant
  - Severe alcohol dependence/withdrawal syndrome which, in the investigator’s judgement, is clinically significant and risks the patient’s safety
  - Acute hepatitis taken as clinical jaundice on examination and/or blood bilirubin level >normal range for local reference criteria or aspartate aminotransferase, alanine aminotransferase (>3× the upper limit of the normal range); or hepatic insufficiency (taken as > 3 times the upper limit of the normal range)
2. History of allergic or adverse reactions to MET, BUP, or the proprietary ATRIGEL delivery system for Sublocade®
3. Clinically significant or uncontrolled mental health problems (including but not limited to psychosis, bipolar disorder, schizoaffective disorder) and/or history or evidence of organic brain disease or dementia that may compromise safety or compliance with the study protocol
4. Current (past 30 day) suicide plan or suicide attempt in the past 6 months
5. Current criminal justice involvement with legal proceedings (not including current probationary supervision) and, in the opinion of the investigator, is expected to fail to complete the study protocol due to re-incarceration or relocation from the centre’s catchment area
6. Currently taking oral or depot naltrexone therapy or enrolment in any form of naltrexone therapy within 90 days prior to study screening
7. Any contraindication to BUP or MET

### BUP-XR dosing and administration

BUP-XR (RPB6000; Sublocade ®; the active investigational medicinal product in this study) is a 200-mg/mL solution of BUP base in the proprietary ATRIGEL delivery system. ATRIGEL is a biodegradable polymer dissolved in a biocompatible solvent non-medicinal product. BUP-XR will be administered by subcutaneous injection into the participant’s abdominal adipose tissue by trained site investigators, medical practitioners, and nurses. The area for administration will be between the transpyloric and transtubercular planes (i.e. below the waistline and above the hip bone in the region where the body curves at the side to about 5 cm from the middle of the abdomen).

To minimise risk of irritation, four different injection points will be used during study treatment. From patient’s perspective, the body area for the injection site will rotate sequentially usually starting in the following sequence: right upper, left upper, left lower, and right lower. In each area, a needle insertion point will be selected with adequate amounts of subcutaneous tissue; no excessive pigment, nodules, lesions, or hair or areas with brawny or fibrous subcutaneous tissue; a location that is not likely to be rubbed or compressed by clothing. Prior to injection, a cold press may be administered for up to 10 s.

In EXPO, dosing will commence with a 300-mg loading dose administered for the first 2 months, followed by a 100-mg maintenance dose for 4 months to the primary endpoint. The scheduled dosing interval will be 28 days, with a minimum interval of 21 days between the two loading doses to provide increased attendance flexibility for the participant (Table 1). If the participant misses a scheduled maintenance dose, no adjustment in dose will be required, as long as they receive BUP-XR within 60 days of their last injection. If the participant does not receive BUP-XR within 60 days of their last injection, they will not be withdrawn from the study treatment, but an assessment by the CI or PI will be required to determine the starting dose to resume treatment (Table 1).

**Table 1:** Dosing schedule for BUP-XR in the study

Dose	Scheduled day	Visit	Window (day)	Dose
1	1	Baseline	-	300mg (loading)
2	28	Week 4	21–42	300mg (loading)
3	56	Week 8	54–70	100mg or 300mg
4	84	Week 12	82–98	100mg or 300mg
5	112	Week 16	110–126	100mg or 300mg
6	140	Week 20	138–168	100mg or 300mg
7>	168>	Week 24> (every 28 days)	Up to 42 days since previous dose	100mg or 300mg

The second 300-mg loading dose of BUP-XR will be given after a minimum of 21 days. The 100-mg maintenance dose can be administered up to 2 days ahead of the schedule (i.e. 26 days since the last injection). Unexpected delays of up to 14 days are not anticipated to have any clinical impact on treatment response, so all subsequent doses can be given up to 14 days after the 28-day scheduled interval (i.e. to 42 days)

*BUP-XR*, extended-release injectable buprenorphine (RBP6000; Sublocade ®)

Applying principles of measurement-based care, the aim will be to maintain a 100-mg monthly dose if the participant abstains from non-medical opioids, has no clinically significant opioid withdrawal symptoms, has no distressing craving for opioids, and is satisfied with their current dose and wishes treatment to continue at this level. However, as a guide, the maintenance dose will be *increased* from 100 to 300 mg if the participant:

1. Reports persistent use of non-medical opioids
2. Experiences opioid withdrawal symptoms
3. Experiences distressing cravings for opioids
4. Has no adverse events related to the 100-mg dose (e.g. sedation or lethargy, persistent headaches, nausea) and there are no other safety concerns
5. Considers the 100 mg to be too low and they request it to be increased

Also as guide, the maintenance dose will be *decreased* from 300 to 100 mg if the participant:

1. Experiences dose-related adverse events (e.g. sedation or lethargy, persistent headaches, nausea)
2. Considers that 300mg is ‘too high’ and they would like it reduced
3. Is seeking to reduce their dose so they can start a withdrawal taper
4. There are no clinical concerns that dose reduction would lead to deterioration with respect to the participant’s substance use and health status

On the basis of patient report and clinical judgement of the risks and benefits, ‘rescue dosing’ with BUP-SL can be provided at any point after the first dose of BUP-XR. This will be recorded and reported as concomitant medication.

### BUP-XR beyond endpoint

Participants allocated to BUP-XR will be able to receive continued BUP-XR past the EXPO endpoint for the duration of the study if there are no safety concerns (including a negative pregnancy test) and a Participant Consent Form (PCF) is completed. During continued treatment, liver function tests will be done approximately every 6 months, or as clinically indicated.

### SOC medication dosing and administration

The active oral comparator medications in EXPO are the two SOC medications for OUD: BUP-SL and MET. BUP-SL tablets are available as 0.4 mg, 2 mg, and 8 mg strengths (usual dose range: 8–24 mg/day). MET is 1mg/1ml oral solution (usual dose range: 60–120 mg/day). Both medications follow the same dispensing regimen. BUP-NX and BUP-ESP may also be used. BUP-NX tablets are available as 2 mg/0.5 mg, 8 mg/2 mg, and 16 mg/4 mg strengths (usual dose range: 8–24 mg/day). BUP-ESP is available in 2 mg/0.5 mg and 8 mg/2 mg strengths (usual dose range: 8–18 mg/day). BUP-NX and BUP-ESP will be classified as BUP-SL for reporting.

EXPO centres will adhere to UK clinical guidelines with SOC medication treatment commencing with directly observed dosing in a community retail pharmacy, followed by provision of patient self-administered ‘take home’ doses according to satisfactory clinical response (adherence and negative opioid using drug screen tests). The SOC dosing level will be adjusted for the patient according to clinical response and their willingness. In all centres, the choice of SOC medication will be determined by local hospital pharmacy policy, assessment, and medication management policies. Reflecting usual clinical practice, participants in the SOC arms may transition from BUP-SL to MET and from MET to BUP-SL in the course of their treatment.

### Transport, storage of IMP, and SOC medication

BUP-XR must be stored in a secure environment, maintaining a temperature between 2 and 8°C. Appropriate storage conditions (for pharmacy and clinic fridges) will be ensured by completion of temperature monitoring logs. For EXPO, commercial USA licenced stock of BUP-XR will be manufactured under contract to Indivior by Albany Molecular Research Inc. (Burlington, MA). Sublocade stock will be imported into the UK to Sharp Clinical Services. Sharp will then distribute to each centre. Dispensed BUP-XR will be transported to each centre for administration, using an appropriate transit method to maintain the cold-chain, and recorded on approved documentation for audit. On arrival at the centre, BUP-XR will be checked and documented before being placed in a locked room in a temperature controlled, locked, and monitored pharmaceutical refrigerator. BUP-SL and MET will be stored securely at community pharmacies with no requirement for temperature or accountability records to be monitored centrally within EXPO.

### Psychosocial intervention

At the South London site, EXPO will include a psychosocial intervention (PSI). The PSI was developed by EXPO investigators [33, 34, 39–41]. The PSI represents a point of departure from the traditional manual-guided psychological therapy approach in the field in which there is proscription of a sequence of specific interventions offered to the patient. The PSI is a case formulation-driven intervention to develop a working hypothesis between the patient and therapist of how OUD and CUD are maintained and can be addressed.

A non-judgemental, collaborative counselling style [42] is used to encourage participants to set behavioural change goals for drug use and co-occurring psychological disorders. A clinical history is taken, including exposure and response to previous treatments for OUD and CUD. There is a focus on typical and unusual episodes of drug use, including contexts, triggers, physical sensations, elaborated cognition (attention, images, beliefs, appraisals, motivation), coping strategies, actions, and problematic affective and behavioural responses. The PSI will have available the change techniques drawn from the following therapeutic approaches: cognitive behavioural coping and skills training [43]; contingency management (behavioural reinforcement; a total budget of GBP 120 for each participant to motivate abstinence, clinic attendance and recovery activities) [44]; behavioural activation and cognitive therapy methods to treat depression [45]; behavioural psychotherapy for couples to promote relationship stability and abstinence reinforcement [46]; and 12-step facilitation therapy for self-help group attendance [47]. Each PSI intervention is expected to include two or more change techniques.

Sessions with a psychologist will be usually weekly with duration of treatment but will be flexible according to the needs of each participant. A random 5% sample of session recordings per therapist will be independently rated using a scale for rating core and generic psychological skills developed at University College London [48]. The PSI will be reported following the Template for Intervention Description and Replication (TIDieR) checklist [49].

### Discontinuation of treatment

A participant may be discontinued from study medications for any of the following reasons:

1. Safety — including adverse events or significant concomitant illness, injury, or urgent surgeries/procedures that, in the opinion of the CI or PI, are likely to compromise treatment safety or contribute to a deterioration in the patient’s clinical condition
2. Participant request — they will be free to withdraw at any time
3. Sponsor, regulatory agency, or Research Ethics Committee request
4. Pregnancy — if not terminating, the participant will be asked to discuss with the clinician and then continue with BUP-SL or MET or withdraw from medication following usual practice. Participants receiving BUP-XR will not receive further injections and will either receive BUP-SL, MET, or will taper
5. Administrative discharge — due to non-adherence with local clinical policy

In the event of an emergency, or if clinically indicated, a decision to surgically remove the BUP-XR depot (up to 14 days from injection) may be made by the CI or PI, following discussion with the participant. An appropriately skilled medical practitioner will perform the following minor surgical procedure, as follows:

- Palpate of the depot and surrounding area to confirm location
- Cleanse area with antiseptic solution
- Infiltrate area with local anaesthetic
- Cover the area with sterile drape
- Incise the skin up to the subcutaneous tissues with scalpel
- Using blunt and sharp dissection, identify the plane between the depot and surrounding subcutaneous tissues and separate the superficial 25% of the circumference of the depot with blunt dissection
- Gently lift the incised ellipse of skin and depot with forceps
- On removal of the depot, ensure haemostasis and close skin with non-absorbable sutures

Unless the participant withdraws consent, all efforts will be made to collect research data at scheduled study timepoints among those who withdraw from study treatment. There will be a pragmatic focus on collection of the primary outcome in this situation.

### Allocation and stratification

The King’s College London Clinical Trials Unit (KCTU) will programme and independently manage participant randomisation using a secure, password-protected, web-accessed system. The Trial Manager (TM) will allocate randomisation system usernames and passwords to authorised study staff. In the study population, non-medical drug injecting is a prognostic factor for negative treatment outcome [7]. Participants will be stratified by study site (NHS trust and city/town) and current (last 28 days) drug injecting status (yes/no). The randomisation procedure will use stratified random blocks of varying size to ensure even allocation. Participants will be allocated to groups 1 and 2 on a 1:1 ratio in all centres. At commencement of recruitment, participants will be allocated to groups 3 and 4 at the South London site on a 4:1 (in favour of groups 1 and 2) given resource capacity. Once randomised, the system will automatically generate confirmation emails to key staff with the treatment allocation information. At the point of study enrolment, the following clinical pathways will be followed:

1. Participants receiving BUP-SL or MET allocated to continued SOC will receive medication according to the centre’s screening, induction/stabilisation and maintenance dosing, and medication dispensing policy
2. Participants receiving BUP-SL — who are prescribed <8 mg/day — and are allocated to BUP-XR will be given 8 mg/day of BUP-SL for a minimum run-in of 3 days before their first injection; those receiving 8≥ mg/day BUP-SL will receive their first injection without delay (with the last dose of BUP-SL taken 1 day prior)
3. Participants receiving MET who are allocated to BUP-XR will be first converted to BUP-SL following the centre’s clinical procedure; once stabilised, they will require at least 3 days on 8–24 mg of BUP-SL before they can receive their first injection (their last dose of BUP-SL will be taken 1 day prior)

The target will be for all participants in the BUP-XR arms to receive their first injection within the first week following randomisation. Participants will receive payment (weighed by research burden) to offset time and any travel costs to attend each centre to complete research measures.

### Research assessments

EXPO will use the following measures recorded during participants’ visits to each centre (see Table 2 for administration timing):

**Table 2:** SPIRIT schedule of enrolment, intervention, and assessments

	Study week																	
Measure	B	R	1	2	4	6	8	10	12	14	16	18	20	22	24	W	E	52
Consent and screening	X																X	X
SCID-5-RV	X								X						X			X
LFT	X				X				X						X		X	
BUP-XR			X		X		X		X		X		X				X	
SOC (BUP-SL or MET)	X		X	X	X	X	X	X	X	X	X	X	X	X	X			
TLFB	X		X	X	X	X	X	X	X	X	X	X	X	X	X			X
ALC-QFM	X														X			
VAS-N (H/C)	X				X		X		X		X		X		X	X		
VAS-W (H/C)	X				X		X		X		X		X		X	X		
CEQ-F (H/C)	X				X		X		X		X		X		X	X		
MoCA	X								X									
QIDS-SR	X				X				X						X			
DERS-SF	X				X				X						X			X
WSAS	X				X				X						X			
PHQ-15																		X
PHQ-4																		X
EQ-5D-5L	X								X						X			
OSTQOL																		X
KCF					X				X						X			
Qualitative interview #1															X			
Qualitative interview #2																		X
ADSUS	X								X						X			
SURE					X				X						X			
PRO-S	X																	
PRO-I					X				X						X			
ADAPT	X				X				X						X			
CGI-S	X																	
CGI-I					X				X						X			
UDS				X	X	X	X	X	X	X	X	X	X	X	X	X		X
CONMED			X	X	X	X	X	X	X	X	X	X	X	X	X			
Adverse event log			X	X	X	X	X	X	X	X	X	X	X	X	X			
Research payments	X		X	X	X	X	X	X	X	X	X	X	X	X	X			X

*B* baseline, *R* randomisation, *W* withdrawal, *E* extended BUP-XR study treatment; 52 interview #2, *SCID-5-RV* Structured Clinical Interview for DSM-5 disorders — research version, *LFT* liver function tests, *BUP-XR* extended-release buprenorphine, study IMP, *SOC* standard-of-care, study comparator, *TLFB* TimeLine Follow-Back, calendar-prompt interview, *ALC-QFM* alcohol — quantity, frequency, and maximum consumption, *VAS-N (H/C)* visual analogue scale of perceived need for heroin and cocaine, *VAS-W (H/C)* visual analogue scale of perceived want for heroin and cocaine, *CEQ-F (H/C)* Craving Experiences Questionnaire for heroin and cocaine, *MoCA* Montreal Cognitive Assessment, version 7.1 (baseline) and 7.2 (follow-up), *QIDS-SR* Quick Inventory of Depressive Symptomatology — Self-Report, *DERS-SF* Difficulties in Emotion Regulation Scale — Short Form, *WSAS* Work and Social Adjustment Scale, *PHQ-15/4*; Patient Health Questionnaire (15 items and 4 items), *OSTQOL* Opioid Substitution Treatment Quality of Life scale, *KCF* Clinical Keyworker Contact Form; *Qualitative interview (1)*, conducted at South London among participants allocated to BUP-XR, BUP-XR with PSI and BUP-SL or MET with PSI, and in West-Midlands England, North-East England, and Tayside among participants allocated to BUP-XR; *Qualitative interview (2)*, conducted at South London and North-East England, among participants receiving longer-term BUP-XR treatment; *ADSUS* Adult Service Use Schedule, *SURE* Service User Recovery Evaluation, *PRO-S/I* patient-reported outcome — severity and improvement, *ADAPT* Addiction Dimensions for Assessment and Personalised Treatment, *CGI-S/I* Clinical Global Impression — severity and improvement, *UDS* urine drug screen, *CONMED* concomitant medication, reviewed at weeks 4, 12, and 24; *Research payments* (baseline, 24, and ~52-week qualitative interview is GBP 20 to offset time and cover travel and transferred to prepaid card; clinical attendance at weeks 1, 2, 4, 8,10,12, 16,18, and 20 to complete research measures is GBP 10; brief completion of research measures at weeks 6, 14, and 22 is GBP 5

#### Standardised clinical interviews with participants

- *Structured Clinical Interview for DSM-5 disorders — research version* (SCID-5-RV) [50]. The SCID-5-RV contains a checklist of 11 symptoms (presence or absence) to diagnose (in the present study) the severity of current OUD and CUD (mild: 2–3 symptoms; moderate: 4–5; severe: ≥6). The American Psychiatric Association’s definition for remission will be applied at 3-month and 6-month follow-up (i.e. without OUD or CUD criteria [except] craving, using the ‘on maintenance therapy’ specifier as appropriate). The CI and PI can delegate the administration of this instrument to a suitably trained health care professional at all visits after screening.
- *TimeLine Follow-Back* (TLFB) [51]. The adapted TLFB procedure is a field-standard, calendar-prompt, structured interview that will be administered at each study visit and/or phone contact to record each day the participant reports having used and not used non-medical opioids, cocaine, and benzodiazepines. Completion of the TLFB yields a continuous record for the primary outcome.
- *Alcohol consumption — frequency, quantity, and maximum consumption* (ALC-FQM). For the past 28 days, the ALC-FQM will record the number of drinking days, typical quantity of alcohol consumed on a drinking day, and maximum consumption on any 1 day using items from the *Treatment Outcomes Profile* (TOP) [52]. The TOP is the standard national instrument for monitoring the outcomes of alcohol use disorder treatment in England.
- *Visual analogue scale (VAS) for the perceived need and want for non-medical opioids and cocaine (VAS-N and VAS-W)* [53, 54]. Each VAS scale will be a 10-cm line (rated 0–100; anchored at one end by the absence of the subjective state and at the other end by its maximal intensity). The participant is asked to mark a point on the line to provide a continuous (interval) rating of peak strength of needing and wanting non-medical opioids and cocaine in the past 7 days.
- *Craving Experience Questionnaire — frequency version (CEQ-F)* [55]. From the Elaborated Intrusion theory of desire [56], the CEQ-F is an 11-item rating scale that measures the frequency of intensity, imagery, and intrusiveness aspects of craving for non-medical opioids and cocaine in the past 7 days. Each item is rated on an 11-point scale (not at all–constantly; 0–10). The total score ranges from 0 to 110.
- *Montreal Cognitive Assessment* (MoCA) [57]. The MoCA is a brief screening instrument for mild cognitive impairment (i.e. attention, concentration, working memory, visuo-constructional skills, and conceptual thinking). A score of ≥26 is considered normal range functioning. Version 7.1 will be administered at baseline. The alternate form (version 7.2) will be administered at follow-up to decrease the risk of learning effects.
- *Quick Inventory of Depressive Symptomatology — Self-Report* (QIDS-SR [58]). The QIDS-SR is a 16-item measure of depressive symptom severity domains (i.e. low mood, concentration, self-criticism, suicidal ideation, interest, energy/fatigue, sleep disturbance, appetite/weight change, and psychomotor agitation/retardation) in the past 7 days. Each item is scored 0–3. The total score ranges from 0 to 27.
- *Difficulties in Emotion Regulation Scale — Short Form* (DERS-SF [59]). The DERS is an 18-item self-report scale of emotional dysregulation. It has six subscales: non-acceptance of emotional responses, difficulty engaging in goal-directed behaviour, impulse control difficulties, lack of emotional awareness, limited access to emotion regulation strategies, and lack of emotional clarity. The total score ranges from 18 to 90, with higher scores reflecting greater emotion dysregulation.
- *Work and Social Adjustment Scale* (WSAS [60];). The WSAS is a 5-item scale that measures the extent that clinical problems (here OUD) have impaired work tasks, home management, social leisure activities, private leisure activities, and close relationships in the past 2 weeks (each item rated on a 0–8 rating scale). The total score is interpreted as 1–10 (mild impairment), 11–20 (moderately severe impairment), and 21–40 (severe impairment).
- *Patient Health Questionnaire-15* (PHQ-15 [61];). The PHQ-15 is a scale of somatic symptoms in the past 4 weeks. For the past 4 weeks, the participant will be asked to rate the severity of symptoms on a 3-point scale (0, not bothered at all, to 2, bothered a lot). The total score ranges from 0 to 30.
- *Patient Health Questionnaire-4* (PHQ-4 [62];). The PHQ-4 is a brief screening scale of psychological distress in the past 2 weeks (score range 0–12).
- *EQ-5D-5L* [63] is a brief generic scale recording mobility, self-care, usual activities, pain/discomfort, anxiety/depression — each of these dimensions has five levels: no problems, slight problems, moderate problems, severe problems, and extreme problems (score: 1–5). These responses generate health profiles from which health utilities can be calculated for economic evaluations. This rating scale also includes an EQ VAS (10-cm line, rated 0–100) with the following endpoints: ‘the worst health you can imagine’ and ‘the best health you can imagine’.
- *The Opioid Substitution Treatment Quality of Life scale* (OSTQOL [64];). The OSTQOL is a 38-item instrument assessing quality of life specific to patients in BUP-SL and MET treatment across six subscales: personal development, mental distress, social contacts, material well-being, opioid substitution treatment, and discrimination. Each item is scored 0–4. The total score ranges from 0 to 156.
- *Clinical Keyworker Contact Form* (KCF). The KCF is a study devised measure that summarises (1) the number of short (<30 min) and longer (>30 min) discussions between the participant and their keyworker in the past month; (2) a summary checklist of issues discussed during these contacts in the past month — medication prescriptions, drug use, alcohol use, tobacco/nicotine use, physical health, mental health, finance/welfare benefits, housing, legal, relationships, childcare, education and training, recreation, and other topics; and (3) whether there was a review of the participant’s care plan, progress towards a treatment goal, and setting of a new goal.
- *Adult Service Use Schedule* (ADSUS [65];). The ADSUS is a structured interview to record patient-level use of primary care services; emergency department and hospital care; services provided by local authorities (including accommodation, day care, and drop-in centres); and personal costs in terms of days off work, out-of-pocket expenses, and time spent seeking healthcare. The ADSUS has been used for studies of OUD treatment. Information on services received at the centre and other services will also be recorded from the electronic patient record.

#### Patient-reported outcomes and evaluations — OUD/recovery specific

- *Patient-reported outcome — severity and improvement* (PRO-S; PRO-I [66]). The PRO-S is a single 7-point rating of the severity of OUD at baseline. The PRO-I is a single 7-point rating of the extent of improvement in OUD.
- *Service User Recovery Evaluation* (SURE [67];). The SURE is a 21-item measure of perceived ‘recovery status’ in the following domains: substance use, material resources, outlook on life, self-care, and relationships (total score range: 21–63 with a higher score indicating greater perceived recovery status).
- *Qualitative exit interview (#1 and #2)*. Interview #1 is a semi-structured, topic-guided, audio-recorded, qualitative interview (based on the domain structure of the ADAPT) conducted at the study endpoint at the South London, West-Midlands England, North-East England, and Tayside centres. Interview #2 is a qualitative interview based on the structure of the OSTQOL with additional assessment measures (see Table 2) conducted at the South London and North-East England centres among participants who consent to continued BUP-XR treatment beyond the endpoint. Both interviews will be recorded on locally approved devices and transcribed verbatim.

#### Clinician-reported and observed measures

- *Addiction Dimensions for Assessment and Personalised Treatment* (ADAPT) **[**68]. The ADAPT is a 14-item rating scale that assesses OUD severity (three items, score range 0–5), coexisting problem complexity relating to health, personality, relationships, risk to self and others, housing, and finance (seven items; score range 0–15), and recovery capital (four items; score range 0–11). The CI and PI can delegate the administration of the ADAPT to a suitably trained health care professional at all visits after screening.

- *Clinical Global Impression — severity and improvement* (CGI-S, CGI-I) [67]. The CGI-S is a single 7-point rating of the severity of opioid-related problems at baseline. The CGI-I is a single 7-point rating of the extent of improvement in opioid-related problems. The CI and PI can delegate the administration of these ratings to a suitably trained health care professional at all visits after screening.
- *Urine drug screen (UDS; detection sensitivity: opioids: 2000ng/ml; cocaine and benzodiazepines: 300ng/ml [72-h detection window])*. A tamper-proof, instant result, immunoassay device (e.g. E-Z Split Key Cup; www.concateno.com) will screen for recent use of opioids, cocaine, and benzodiazepines. The device uses a control line and a temperature sensor (required range: 92–96° F) to indicate that a valid sample has been collected. The UDS product for the study also includes measurement of BUP and MET (providing a proxy indicator of medication adherence).
- *Liver function tests (LFT; laboratory serum/blood test*). For safety, participants will be screened for liver function (as defined in the inclusion/exclusion criteria) after consent either by conducting an LFT test following each site’s local laboratory procedure or accessing this information from the participant’s hospital medical records if a prior LFT test result has been done and recorded within 12 weeks from the date of screening. If the participant does not have their bloods taken post-randomisation for any reason, they may continue in the trial at the clinical judgement of the PI or sub-investigator. Participants randomised to BUP-SL or MET will have LFT testing according to their local standard of care.

The schedule of assessments for the study is summarised in Table 2.

Source Data Worksheets documenting containing the research assessments and data collection points will be provided to each site by the TM. All baseline and follow-up data will be entered online using InferMed MACRO — an online electronic data capture (EDC) system (www.infermed.com). This system is regulatory compliant (GCP, and the EC Clinical Trial Directive). An electronic case report form (eCRF) using the MACRO EDC will be programmed by KCTU and hosted on a dedicated secure server. The eCRF system will have full audit trail, data discrepancy functionality, and database lock functionality and supports real-time data cleaning and reporting. The TM will request usernames and passwords to any new researchers (only those authorised by the TM will be able to use the system).

### Primary outcome measure

The primary outcome is days of abstinence from all non-medical opioids. With a 1-week measurement grace period from randomisation, this is the count of days abstinent between days 8 and 168 (i.e. weeks 2–24; 161 days), combined with up to 12 UDS tests for opioids (thereby providing biological verification of 36 of the 161 days in the outcome measure). If a UDS test result is positive for opioids, then the day of the test and 2 days prior will be recorded as positive for opioids, thereby overriding a discrepant report on the TLFB.

### Secondary outcome measures

EXPO has the following secondary outcome measures:

1. Safety measured by all adverse event reporting
2. Time (days) enrolled in the study treatment (retention) to week 24
3. Days abstinent from cocaine and illicit/non-medical benzodiazepines during weeks 2–24 (combining TLFB and UDS data)
4. Craving (need and want) for heroin and cocaine (VAS-N and VAS-W)
5. Craving (elaborated experience) for heroin and cocaine (CEQ-F[H] and CEQ-F[C])
6. OUD and CUD DSM5 status measured by SCID-5-RV
7. Clinician rating of severity, complexity, and recovery strengths by ADAPT
8. Clinician rating of global impression (CGI-I anchored on baseline CGI-S)
9. Difficulties in Emotion Regulation — Short Form (DERS-SF)
10. Patient report of depression symptoms (QIDS-SR)
11. Patient report of work and social adjustment functioning (WSAS)
12. Patient evaluation of OUD recovery (SURE)
13. Patient report of OUD improvement (PRO-I anchored on baseline PRO-S)
14. Cognitive function (MoCA)
15. Alcohol use (typical quantity frequency and maximum consumption; ALC-QFM)
16. Among participants enrolled in longer-term BUP-XR treatment, the following measures will be administered: heroin, cocaine, and illicit/non-medical benzodiazepine use in past 90 days (TLFB; UDS); OUD and CUD remission status (SCID-5-RV); somatic symptoms (PHQ-15), emotion regulation (DERS-SF), depression and anxiety symptoms (PHQ-4) and quality of life (OSTQOL)

### Sample size

Informed by the DELTA2 guideline [69], sample size calculations were strategic to ensure a reliable estimate of the treatment effect. With an estimate assumed to be equivalent in each phase and informed by a study of SOC maintenance medication for OUD and PSI conducted at the EXPO co-ordinating centre (ARC Trial; ISRCTN69313751) [29], the required number of participants was estimated from the requirements of a Poisson regression model with a baseline rate of 0.6 and with an expected — and clinically meaningful — 23% target difference in the count of days of abstinence from all non-medical opioids during 161 days after randomisation.

To obtain 90% power, with alpha at 5% and with 15% inflation for attrition, a target total of 304 participants will be needed for the group 1 (BUP-XR; *n* = 152) versus group 2 (SOC; *n* = 152) comparison, and 300 participants for the group 3 (XR-BUP and PSI; *n* = 150) versus group 4 (SOC and PSI; *n* = 150) comparison. The Statistical Analysis Plan (SAP) will present a sensitivity check on this power calculation on the assumption of a greater group response.

The strategy to achieve adequate participant enrolment to reach the target sample size will be based on periodic review of clinical caseloads in each site to identify patients likely to be eligible and who may be interested in taking part and also by providing information about the study at any appropriate point in the screening process for OUD treatment.

### Analysis plans

#### Statistical analysis plan

The SAP will describe the steps for the analysis of the primary and secondary outcomes. It will be approved by the independent trial committees and published on the Open Science Framework (www.osf.io) before data-lock. The senior statistician will be blinded. The junior statistician will be unblinded so that reports can be prepared. The research and clinical team will also be unblinded. Findings will be reported following the Consolidated Standards of Reporting Trials [70]. Final statistical command code will be published on the OSF. There are no interim analyses and specified trial stopping rules.

The analysis will be conducted in STATA or R and the analysis will follow the intention-to-treat (ITT) principle (i.e. all patients will be analysed in the group to which they will be allocated) with alpha set at 5% (two-tailed). The distributions of scale and count measures may be non-normal (skewed) — therefore, test statistics and effect sizes may be computed following appropriate transformation (e.g. natural log to obtain a geometric mean).

A maximum-likelihood multiple imputation approach will be used for the management of missing data with a sensitivity comparison to the complete case dataset. Pooling data from all EXPO centres, a mixed-effects multivariable regression model will be done for the analysis of the primary outcome, with covariables (sex, age, drug injecting status, site, and baseline score of the outcome measure), and a site-varying random intercept. The medication preference factor may be included through interaction tests because it is expected that some participants will have a preferred OUD SOC medication due to past or current exposure. The cumulative distribution function of the primary endpoint will also be plotted for comparison purposes. Other graphical representations may be used for treatment effect visualisation. For the primary outcome, the following sub-group analyses will also be done:

1. Using cocaine (yes/no)
2. Length of time in treatment (less than 1 month/1 month or longer)
3. Benzodiazepine use past month to admission (yes/no)
4. CGI-S (mild/severe)

Analyses of secondary outcomes will proceed using the same stratification and covariates as defined for the primary analysis model using an appropriate linear (continuous measures) or logistic (binary or ordinal measures) regression framework. An exploratory mediation analyses — including VAS-N/W, CEQ-F, QIDS-SR, MoCA, and WSAS baseline and follow-up measures — will be implemented in the counterfactual (causal inference) framework and will include a baseline covariables and the treatment/mediator interaction.

#### Health Economic Analysis Plan

The Health Economic Analysis Plan (HEAP) [71] will be approved by the trial committees and published on the OSF before data-lock. The HEAP will describe the analytic steps for a cost-effectiveness analysis. This will consider patient QALYs and costs from a broad societal perspective including NHS and personal social services, productivity losses (including time off work because of illness), and criminal activity. This will be based on an incremental analysis of the mean costs and QALYs for BUP-XR versus BUP-SL or MET. The analysis will be reported following the Consolidated Health Economic Evaluation Reporting Standards [72].

EXPO participants’ direct and indirect costs will be estimated from responses to the ADSUS, and the KCF will be used to record the clinical team’s direct and indirect time working as part of the trial. This will ensure missing data on important cost drivers are reduced to a minimum. Unit costs will be obtained from routine hospital data (NHS reference costs) and other resources such as the British National Formulary for medicines, and the unit costs of health, social care, and criminal justice compiled by the University of Kent’s Personal Social Services Research Unit. Indirect costs will be valued using the human-capital method, based on the average annual earnings data by sex and age group obtained from the Office for National Statistics.

QALYs will be calculated from EQ-5D-5L scores and by applying the method specified by the National Institute for Health and Care Excellence. The economic analysis has no implications for the sample size calculation. The number of QALYs experienced by each participant will be calculated as the area under the curve, using the trapezoidal rule, and adjusted for baseline [73]. Total costs and QALYs will be used to calculate the incremental cost-effectiveness ratio of BUP-XR versus BUP-SL and MET. Data that are assumed missing at random will be imputed using multiple imputation by chained equations. Non-parametric bootstrapped 95% central ranges for items of resource use, costs, and QALYs will be estimated (using 10,000 replicates).

A range of one-way sensitivity analyses will be conducted to test whether, and to what extent, the incremental cost-effectiveness ratio is sensitive to key assumptions in the analysis (e.g. unit prices). Multivariate sensitivity analyses will be applied where interaction effects are suspected, and the joint uncertainty in costs and benefits will be considered through application of bootstrapping and estimation of cost-effectiveness acceptability curves [74]. Alternative scenarios will be specified including consideration of a narrower cost perspective (NHS ± personal social services) to enable comparison with the NICE threshold range of GBP 20,000–30,000 per QALY.

### Longer-term data linkage

After completing and reporting the analyses of the primary and secondary analyses, there is a planned longer-term exploratory analysis of outcomes at 3 and 6 years following randomisation using linked UK registry data. Subject to patient consent and approval from the Office for Health Improvement and Disparities (formally Public Health England), the Ministry of Justice, and NHS Digital, EXPO participant data will be linked to the following:

1. National Drug Treatment Monitoring System (NDTMS) to include, but not limited to, (a) history of treatment recorded on NDTMS, (b) number of episodes and time enrolled in community and prison setting treatments, and (c) treatment status at exit(s)
2. NHS hospital episodes statistics (HES) contacts with inpatient and outpatient hospital services (as captured by the various HES databases)
3. NHS Digital to include (a) incident and date of mortality, (b) cause of mortality, (c) involvement of alcohol or drugs, and (d) location of death. Case definitions will include ‘Mental and behavioural disorders due to drug use’ (ICD-10 codes: F11-F16, F18, F19) and an opioid was mentioned on the death certificate; or to any of the following: ‘Accidental poisoning by drugs, medicaments and biological substances’ (X40-X44); ‘Intentional self-poisoning by drugs, medicaments and biological substances’ (X60-X64); ‘Assault by drugs, medicaments and biological substances’ (X85); and ‘Poisoning by drugs, medicaments and biological substances, undetermined intent’ (Y10-Y14), where any controlled drug and an opioid was mentioned (and potentially referring to the same drug, such as heroin)
4. Police National Computer (PHC) to include (a) lifetime convictions history and profile to study enrolment and (b) change in number of offence types [where a person was charged, then subsequently proven guilty and either convicted, cautioned, reprimanded, or warned] for a 2-year period before randomisation and follow-up

This analysis of extracts from NDTMS, HES, NHS Digital, and the PNC will be implemented subject to resources and requiring protocol amendment and analysis plans.

### Oversight, monitoring, and dissemination

An independently chaired Trial Steering Committee (TSC) and Data Monitoring Committee (DMC) will oversee this Clinical Trial of an Investigational Medicinal Product, and its integrity, recruitment procedures, research measures and their completion, and the data analysis. These committees will include members with addiction service delivery, commissioning, service management, and patient and public involvement (PPI) expertise. The Trial Management Group will be responsible for the day-to-day running of the study and members will attend meetings of the oversight committees. After approving the protocol, the TSC and DMC will meet approximately two to four times each year. The King’s Health Partner’s Clinical Trials Office (KHP-CTO) will monitor EXPO centres every 14–18 weeks (but can be increased/decreased). While there are no trial stopping rules, the study may be prematurely discontinued by the sponsor, or for reasons reported by the chair of the DMC to the chair of the TSC.

#### Safety and adverse event reporting

The Reference Safety Information for all information pertaining BUP-XR will be the Investigator’s Brochure (IB). The Summary of Product Characteristics (SmPC) will be the reference document for SOC medication. During the study, adverse events will be defined as follows:

1. Adverse event — will be any untoward medical occurrence in a subject to whom a medicinal product has been administered including occurrences which are not necessarily caused by or related to that product
2. Adverse reaction — will be any untoward and unintended response in a participant to an investigational medicinal product which is related to any dose administered to that participant

Unexpected adverse reaction — will be an adverse reaction the nature and severity of which is not consistent with the information about the medicinal product in question set out in the SmPC or the IB

3. Serious adverse event (SAE) — a serious adverse reaction or suspected unexpected serious adverse reaction — will be any adverse event, adverse reaction, or unexpected adverse reaction, respectively, that results in death, is life-threatening, required hospitalisation or prolongation of existing hospitalisation, results in persistent or significant disability or incapacity, and consists of a congenital anomaly or birth defect
4. Important medical events (IME) —will not be immediately life-threatening or result in death or hospitalisation but may jeopardise the patient or may require intervention and will be considered serious. Although not an SAE, any unplanned pregnancy will be reported as an IME.

All clinical investigators in the study will be provided with full details of possible adverse medical events that may result from study medication. Clinicians will report, and the PI will assess, each adverse event for seriousness, causality (definite, probable, possible, remote, none), and intensity (mild, moderate, and severe). All serious adverse events will be promptly reported to the study sponsor no later than 24 h after the research team becoming aware of the event. The CI (or a doctor nominated by the CI) will review every event within one working day of the SAE form being received and determine whether the event was expected or unexpected. The CI may upgrade the causality of an event without PI agreement.

#### Confidentiality

The CI will have overall responsibility for the trial dataset, supported by the oversight committees, and the co-clinical lead investigator (CCLI) will act as custodian for the trial data under the General Data Protection Regulations. Only the CI, CCLI, KCTU, EXPO statisticians, and EXPO heath economists will have access to the final trial dataset. Participant data (as defined by the Data Protection Act 2018) will not be disclosed to the funder or the sponsor — except where this is required to satisfy safety monitoring — and confidentiality of participant information will be assured through the following adherence:

1. All patient data including audio recordings will be assigned a unique numeric identifier and stored on a password-protected computer
2. All study data will be stored in line with the Medicines for Human Use (Clinical Trials) Amended Regulations 2006
3. All study data will be archived in line with the Medicines for Human Use (Clinical Trials) Amended Regulations 2006 (and as defined by the sponsor’s archiving policy and procedure)

#### Dissemination

Findings from the study will be communicated as reports to peer-reviewed scientific journals, medical conferences, and other scientific meetings, and in target ways appropriate for service user, public, and professional audiences.

## Discussion

The opioid agonist/partial agonist medications BUP-SL and MET are first-line, evidence-based SOC, but not all patients are able to adhere and derive benefit. EXPO is the first randomised controlled trial of BUP-XR versus SOC and the first study to contrast BUP-XR and SOC with personalised PSI.

The study has several strengths. Firstly, it has patient and public engagement; is well-powered and pragmatic, with a protocol open to as many members of the target population as possible in well-established specialist NHS services in areas with a high prevalence of OUD; and implemented under routine clinical conditions. This will increase the likelihood that the findings from the study generalise to NHS services in England and Scotland. If the study secures evidence for the relative effectiveness of BUP-XR over SOC, this will have substantial implications for policy and clinical practice in the UK and elsewhere.

Secondly, the primary outcome is a well-defined, clinically meaningful and combines patient report and biochemical measure. The collection of outcome measures is timed to coincide with routine clinical follow-up as part of efforts to minimise loss to follow-up. Thirdly, EXPO builds on work to develop and study idiographic, personalised PSI as evaluated in the successful ARC study [30]. If there is evidence that BUP-XR can be enhanced by a personalised PSI, it will be a significant advance for the field and for future research. Fourthly, the included qualitative evaluation should illuminate patient perspectives and provide additional evidence for study treatments above and beyond the primary and secondary clinical outcomes.

Limitations of the study include the relatively short 24-week endpoint. This horizon is commonly used in the field, but further exploratory research (as planned in EXPO) will be needed to determine longer-term outcomes on opioid use. Other research questions — for example the deployment of BUP-XR as a taper for medication discontinuation — will need new protocols.

An integrated approach to assessment, stratified treatment, and continuing care is now gaining momentum in behavioural medicine, where tailoring variables and measurement-based care actions can improve outcomes [75, 76]. With reports targeting high-impact medical scientific journals, and presentations to conferences and Patient and Public Involvement events, we expect that the EXPO study will make an important contribution to this applied clinical orientation for effective treatment of OUD.

### Protocol approval and study status

Approval for the study was obtained from the UK Medicines and Healthcare Product Regulatory Agency on 4 March 2019. The study was registered on the EU Clinical Trials Register on 4 March 2019 (number: 2018-00460-63); https://www.clinicaltrialsregister.eu/ctr-search/trial/2018-004460-63/GB. The protocol and PIS/PCF materials (available from corresponding author) were approved Health Research Authority (IRAS project number: 255522) via the London-Brighton & Sussex Research Ethics Committee (reference: 19/LO/0483) on 14 June 2019. Participant recruitment commenced on 6 August 2019. It is anticipated that the study will complete recruitment in November 2021.

### Protocol and amendments

The latest version of the study protocol is version 5.1 (6 October 2021). There have been seven non-substantial and substantial approved amendments to the protocol, as follows:

Version 1.2 (13 June 2019). Non-substantial amendment: (a) use of rescue BUP-SL after first BUP-XR injection and ongoing (to be recorded as concomitant medication); (b) a surgical procedure guideline for the removal of BUP-XR; and (c) all SARs among participants allocated to BUP-XR to be classified as unexpected and reported as SUSARS.

Version 2.0 (8 January 2020). Substantial amendment: (a) dosing guidance for participants consenting to receive continued BUP-XR after the study treatment endpoint; (b) optional use of cold press before BUP-XR injection; (c) removal of instruction that BUP-XR can be stored at room temperature for up to 7 days prior to administration.

Version 2.1 (9 July 2020). Non-substantial amendment: addition of qualitative interview #1 at South London, West Midlands, Newcastle, and Tayside centres.

Version 3.0 (22 July 2020). Substantial amendment: addition of BUP-NX as this is an SOC medication at Tayside site.

Version 4.0 (30 September 2020). Substantial amendment: (a) addition of BUP-ESP as this is an SOC medications at Tayside site; (b) clarification that participants allocated to SOC arms can be transitioned between BUP-SL and MET following prescribing guidelines in the SmPC and if there are no known allergic, adverse reactions or contraindications.

Version 5.0 (1 June 2021). Substantial amendment: (a) addition of OSTQOL; PHQ-4 and PHQ-15 for continued treatment evaluation; (b) option for research measures recorded during clinic visits at weeks 2, 6, 10, 14, 18, and 22 to be done by telephone (precluding UDS collection) in response to government public health restrictions for COVID-19; (c) clarification that participants can continue in the trial if LFT testing is not taken post-randomisation according to CI, PI, and sub-investigator clinical judgement; (d) clarification that participants can continue in study if LFT testing not done post-randomisation according to CI, PI, and SI clinical judgement.

Version 5.1 (6 October 2021). Non-substantial amendment: expanded description of cost-effectiveness analysis for the health economic analyses.

## Supplementary Information

**Additional file 1: Table S1**. Standard Protocol Items for Randomised Trials (SPIRIT) 2013. SPIRIT Checklist.

**Additional file 2.**

**Additional file 3.**

## Abbreviations

ADAPT: Addiction Dimensions for Assessment and Personalised Treatment
ADSUS: Alcohol and Drug Service Use Schedule
AE: Adverse event
AEL: Adverse event log
ALC-QFM: Alcohol — Quantity, Frequency and Maximum Consumption
ATRIGEL: Proprietary Extended-Release BUP Delivery Technology
BUP: Buprenorphine hydrochloride
BUP-ESP: Buprenorphine (Espranor®)
BUP-NX: Buprenorphine-naloxone (Suboxone®)
BUP-SL: Sublingual (tablet) buprenorphine
BUP-XR: Extended-release buprenorphine (Sublocade®; prev. RBP-60000)
CBT: Cognitive Behavioural Therapy
CEQ-F(H/C): Craving Experience Questionnaire (frequency version) (Heroin/Cocaine)
CGI-S and I: Clinical Global Impression (severity and improvement)
CI: Chief investigator
CCLI: Co-clinical lead investigator
CONMED: Concomitant medication
COVID-19: SARS-Cov-2 virus (2019)
CRF: Case report form
CUD: Cocaine use disorder
DERS-SF: Difficulties in Emotion Regulation Scale — Short Form
DMC: Data Monitoring Committee
DSM-5: Diagnostic and Statistical Manual of Mental Disorders fifth edition
eCRF: Electronic case report form
EDC: Electronic Data Capture
EQ-5D-5L: EurolQol Health Status (5 levels)
GCP: Good Clinical Practice
HEAP: Health Economics Analysis Plan
IB: Investigator’s Brochure
IME: Important medical events
IMP: Investigational Medicinal Product
KCF: Keyworker Contact Form
KCTU: King’s Clinical Trials Unit
KHP-CTO: King’s Health Partners-Clinical Trials Office
LFT: Liver function tests (AST, ALT, albumin, and bilirubin)
MET: Methadone hydrochloride (oral solution)
Mg: Milligrammes
Mg/day: Milligrammes per day
MoCA: Montreal Cognitive Assessment
NICE: National Institute for Health and Care Excellence
OSTQOL: Opioid Substitution Treatment Quality of Life scale
OUD: Opioid use disorder
PHQ-4: Patient Health Questionnaire — anxiety and depression
PHQ-15: Patient Health Questionnaire
PI: Principal investigator
PNC: Police National Computer
PRO-S and I: Patient-reported outcome (severity and improvement)
PSI: Personalised psychosocial intervention
QALY: Quality-adjusted life year
QIDS-SR: Quick Inventory of Depressive Symptomatology
SAE: Serious adverse event
SAP: Statistical Analysis Plan
SAR: Serious adverse reaction
SCID-5-RV: Standard Clinical Interview for Dependence — Research version (DSM-5)
SmPC: Summary of Product Characteristics
SOC: Standard-of-care (medications; in EXPO, these are MET and BUP-SL)
SURE: Substance Use Recovery Evaluator
SUSAR: Suspected unexpected serious adverse reaction
TLFB: TimeLine Follow-Back
TM: Trial manager
TMG: Trial Management Group
TSC: Trial Steering Committee
TOP: Treatment Outcomes Profile
UDS: Urine drug screen
VAS-(N/W): Visual analogue craving rating (need/want)
WSAS: Work and Social Adjustment Scale
