# Supplementary material for: Extended-release pharmacotherapy for opioid use disorder (EXPO): protocol for an open-label randomised controlled trial of the effectiveness and cost-effectiveness of injectable buprenorphine versus sublingual tablet buprenorphine and oral liquid methadone
Source: Trials. 2022 Aug 19;23:697. doi: 10.1186/s13063-022-06595-0 (PMC9389497; doi:10.1186/s13063-022-06595-0)
Supplement: Supplementary file 2 — Additional file 2. [file 13063_2022_6595_MOESM2_ESM.docx]

| **Participant Information Sheet for the EXPO study** |
| --- |

**Overview**

This Participant Information Sheet for the EXPO study tells you about a new treatment for people with opioid (heroin) dependence. EXPO stands for **EX**tended-release **P**harmacotherapy for **O**pioid use disorder (**EXPO**).

The aim of EXPO is to compare a new long-acting maintenance medication called extended-release buprenorphine (BUP-XR) with oral maintenance medications (liquid methadone (MET) sublingual tablet buprenorphine, sometimes called Subutex (BUP-SL)

BUP-XR has the same effect as buprenorphine tablets, but it lasts much longer. It is given at the clinic as an injection of liquid under the skin in the abdominal area (below the ribs and above the hip bone). Its effects last for 1 month. The patient doesn’t need to go to the pharmacy to get BUP-XR – rather they get their next dose a month later at the clinic. BUP-XR was developed in the USA by a company called Indivior (its product name is ‘Sublocade’). This study will be the first time Sublocade has been used in England.

Everyone in this study will receive medication and usual support – there is no placebo. No medication will be withheld.

All the information about participants will be kept confidential and securely protected at all times. We will write summary reports that will not identify anyone.

Taking part in this study is voluntary. This sheet will help you decide whether you want to take part. If anything is not clear, please do ask.

**Your invitation**

We are inviting you to take part in the EXPO study. This is because you are either receiving MET (dose 30mg or less) or BUP-SL (dose 24 mg or less), or you are starting treatment. If you are in treatment and receiving more than 30mg of MET or more than 24mg of BUP-SL, you may be able to take part if you reduce your dose. Your clinic doctor will advise.

EXPO is a type of research study called a ‘randomised controlled trial’. This means that a computer will decide at random whether you receive BUP-XR, or usual medication treatment.

If you would prefer to not take part, you will receive or continue to receive treatment as usual. Before you decide to take part, you need to understand why we are doing this research and what it would involve for you.

Please take time to read the following information carefully. Ask questions if anything you read is not clear or you would like more information.

**Why are we doing this study?**

We are asking whether 6 months of BUP-XR is better than 6 months of daily MET or daily BUP-SL for helping people reduce heroin use. Several clinics around England and Scotland are joining us to answer this question.

If we find that BUP-XR is more effective, then Sublocade’s manufacturer could use data from the study to obtain a licence so that more people can receive BUP-XR.

**What will happen to me if I take part?**

If you would like to take part in EXPO, here is a summary of what will happen:

First, we will ask you to complete a consent form and receive a brief medical examination. If you are breast feeding or pregnant you will not be able to take part in the study. Other checks will include a blood test to assess the health of your liver, unless this is known from a test done within 12 weeks where we have this information on your clinical file.

If there is a sign that it’s not working as normal, you won’t be able to take part but you will receive or continue to receive usual treatment. If you pass the medical screening, there will then be a few questionnaires, asking about you and your life. This stage should be completed in a day or so. If you are taking part in the study, we will let your GP know (with your consent).

If you live in England or Wales, we will ask you if we can collect information from you: your name, date of birth, sex (at birth), home postcode, National Insurance number and NHS number. We will use this information to find out about your use of hospital services, any future drug treatment, work, crime and mortality for the 2 years before and after the study. This is to assess how effective the treatment you’ve received in the study is. We will handle and store this personal information securely. **We will not ask to do this if you live in Scotland. You can find more information about how we’ll do this on pages 6 and 7. You can decide you don’t want us to look for this information, but still take part in the study.**

We will give you a wallet card that explains you are taking part in a study involving specified medications – this is in case you need any other medical treatment.

To answer the question is BUP-XR better than standard medication?, the computer will decide at random whether you will receive:

(1) BUP-XR; or

(2) MET or BUP-SL – either the medication you’re on already or the one you’re seeking or happy to receive.

If you are going to get BUP-XR and you are currently on MET, we will transfer you to BUP-SL for a few days while we check you are comfortable and happy to proceed with your first injection of BUP-XR. **There is more information on how BUP-XR is given on Page 5.**

The EXPO study will last for 6 months. People getting BUP-XR will receive 6 injections of BUP-XR at the clinic. People getting MET or BUP-SL will receive medication from a retail pharmacy.

During the study, we will ask you to visit the clinic on 13 occasions and complete some questionnaires (each taking 20 mins or so). These will ask you about craving, any drug use, your health and mood, and there will be a quick urine drug screen to help us see which treatments are working. We will try to time these visits to be when you were due to attend the clinic anyway. You will receive either £20, £10 or £5; loaded onto a prepaid card, for attending the clinic. The amount will vary depending on the time point of the visit.

During the study, women who are engaging in potentially procreative sex must use contraception: the Pill, condoms, diaphragm or a Nexplanon implant. If you get pregnant during the study, the Chief Investigator will discuss your treatment options.

After the study ends, you can stay in treatment for as long as you need. We will transition people getting BUP-XR to MET or BUP-SL (as you prefer, unless there is a medical reason to select one of these). It may be possible to stay on BUP-XR. We will let you know in advance.

After the study, we will continue to collect research information on EXPO participants from public registries – for example, those recording use of hospital services, and any criminal convictions. We are doing this to see if BUP-XR with or without psychological support helps people in the long term.

**What are the possible risks in taking part?**

Here is a list of things to bear in mind:

It is possible that BUP-XR may affect your liver, so during the study, we will need to continue to check it’s healthy. After the initial liver blood test, we will ask you to have a check at 1 month, 3 months and at 6 months during the study. We may ask you to have your liver monitored more often. If you are not receiving BUP-XR your doctor may discuss the need for liver tests with you, as is normal practice at the clinic.

We will also assess other medications you may be taking to make sure they are safe for you.

For some people, it can be painful while BUP-XR is being injected – but any discomfort will pass quickly. We can apply a cold press to the area about to be injected which can help ease this discomfort.

After receiving BUP-XR, you may notice a small lump under the skin where you had the injection. Do not rub or massage the skin where you were injected as this may irritate your skin and it may mean the medication does not dissolve and work properly. Avoid wearing clothing with belts or waistbands that could rub the area of the injection as this could cause irritation. Over time, the small lump will reduce in size as the medication dissolves.

In the very unlikely event that you have an allergic reaction to the BUP-XR, it can be surgically removed within 14 days of the injection. After that, the medication has dissolved, and it is impossible to remove.

It is possible that filling in some questionnaires may be upsetting. We will offer support to help anyone who experiences this.

**What are the possible benefits of talking part?**

The benefits will include you receiving help for opioid (e.g. heroin or MET) dependence and recovery support. Volunteering for EXPO will help us see if this treatment is effective. We cannot promise the study will help you, but the information we get from the study will help to improve the treatment of people with heroin dependence.

**What will happen to the results of EXPO?**

The results of EXPO will be included in academic publications. These reports will bring together the data for all participants. No information that could be used to identify you will ever be published. You will not be identified in any report/publication.

**Who is organising and sponsoring the research?**

King’s College London (KCL) and South London and Maudsley NHS Trust (SLaM) are the joint sponsors for this study. It is being done in five NHS clinics across England and Scotland.

**What about data security and my personal information?**

Keeping personal information safe and secure has always been extremely important to us. It is important to stress that once the information has been gathered for the trial it will be linked to a research number, not your name, and will be held under strict confidentiality rules. It will only be seen by authorised researchers working on EXPO. All the data collected for the trial (e.g. paper forms, research questionnaires), will be kept securely for 10 years by KCL after which it will be archived. Strict arrangements will be in place to make sure that your personal information is handled securely and protected at all times.

**What are my rights and what happens if there is a problem?**

You can decide not to take part, or you can leave the trial at any time, without giving a reason. If you decide to not take part or leave, your rights to health care and benefits will not be affected. If you have any questions or concerns about the information about you that is being used in the study, please speak to the EXPO research team.

**Will my taking part in the study be kept confidential?**

The researchers and study sponsors take data security very seriously and will safeguard your confidentiality. The researchers will be using information from you and your medical records, acting as what’s called the ‘data controller’. This means we are responsible for looking after your information and using it properly in the study. The sponsors will keep securely identifiable information about you for 10 years after the study.

**Further information and contact details:**

Participants based in England and Wales can obtain general information about research from the NHS Patient Advice and Liaison Service for independent advice (free-phone: 0800 731 2864 (Option 2) or by email at [pals@slam.nhs.uk](mailto:pals@slam.nhs.uk)). Participants based in Scotland can contact the NHS Research Scotland General enquiries line on  0141 9515508 or visit [https://www.nhsresearchscotland.org.uk/](https://eur03.safelinks.protection.outlook.com/?url=https%3A%2F%2Fwww.nhsresearchscotland.org.uk%2F&data=01%7C01%7Cjatinder.bisla%40kcl.ac.uk%7C667f4026d75441f72e5b08d79ff6efbd%7C8370cf1416f34c16b83c724071654356%7C0&sdata=m1Nor%2FPdjBn4WitiL2hFRPiMy%2FmAKgEKjSDyzuoYj3U%3D&reserved=0).You can find out more about how we use your information here: https://www.kcl.ac.uk/research/support/research-ethics/kings-college-london-statement-on-use-of-personal-data-in-research.aspx.

If you prefer, information can be obtained in person from the KCL Data Protection Officer, Mr Albert Chan. His email address is: [info-compliance@kcl.ac.uk](mailto:info-compliance@kcl.ac.uk).

The study’s sponsors, KCL and SLaM, have insurance policies for this study. If you experience harm or injury as a result of taking part, you could be eligible to claim compensation without having to prove that KCL and SLaM were at fault. This does not affect your legal rights to seek compensation. If you are harmed due to negligence in the study, then you may have grounds for legal action. Regardless of this, if you wish to complain, or have any concerns about any aspect of the way you have been treated during the study, you should immediately inform the Chief Investigator. The normal NHS complaint mechanisms are also available to you.

**APPENDIX**

**HOW IS BUP-XR GIVEN?**

BUP-XR is given by a doctor or nurse at the clinic as an injection under the skin in your abdominal area (below your ribs and above your hip bone). Here are some pictures to show what this medication looks like and how it is given.

| **A** | **B** |
| --- | --- |
| 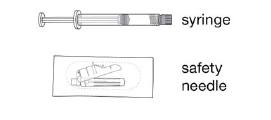 | 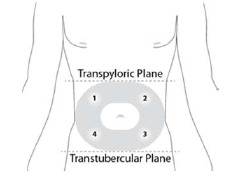  3.LL  4.RL  2.LU  1.RU |
| **C** | **D** |
| 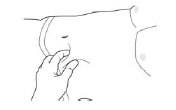 | 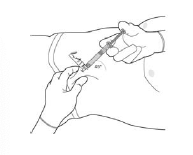 |

Picture **A** shows a pre-filled syringe of BUP-XR. It's a small amount of colourless liquid (sometimes a little amber coloured). The syringe is a single-dose with a safety needle.

Picture **B** shows the injection site. The doctor or nurse will clean the area with a swab.

Picture **C** shows how the skin is pinched to lift it above the muscle.

Picture **D** shows the needle being inserted into tissue.

BUP-XR comes in 2 strengths: 100 mg and 300 mg. You receive 300 mg for your first injection. Then about a month later you’ll receive another 300 mg injection. Then, for the next 4 months you’ll be given a 100mg shot every month (4 injections). Some patients may find that they are using heroin or other opioids and are experiencing a lot of cravings. You can talk to your doctor at the clinic if you think the 300 mg dose would be more effective for you. This dose can be reduced back to 100 mg if you prefer. Your doctor may also give you buprenorphine tablets as a ‘rescue’ if you need it.

**MORE ON DATA CONFIDENTIALITY AND ACCESSING DATABASES**

If you live in Scotland this doesn’t apply but if you live in England and Wales we will ask Public Health England (PHE) to access any information about you on the following databases: (1) services received for drug and alcohol problems; (2) how often you may have visited a hospital outpatient service, or attended accident and emergency, or if you have had to stay overnight in hospital and for how long: NHS Hospital Episode Statistics; and (3) data on mortality (from NHS Digital - Register of births and deaths) and drug and alcohol related convictions and time in prison (from the Ministry of Justice).

Only the EXPO researchers and people in PHE whose job involves collecting and using this data will be able to see it. When we receive information from these databases it will be in a format where it isn’t attached to your name or to any other identifying data. This is because we are only interested in whether EXPO interventions are helping people.

When you agree to take part in any research study, the information about your health and care may be provided to researchers running other research studies in this organisation and in other organisations. These organisations may be universities, NHS organisations or companies involved in health and care research in this country or abroad. Your information will only be used by organisations and researchers to conduct research in accordance with the UK Policy Framework for Health and Social Care Research.

Authorised people from KCL and SLaM and also regulatory organisations may look at your medical and research records to check the accuracy of the research study. Your doctor at the clinic will pass these details to KCL and SLaM along with the information collected about you. The only people in KCL and SLaM who will have access to information that identifies you will be people who need to audit the data collection process. The people who analyse the information will not be able to identify you and will not be able to find out your name, NHS number or your contact details.

Your information could be used for research in any aspect of health or care and could be combined with information about you from other sources held by researchers, the NHS or government. If this information could identify you, it will be held securely with strict arrangements about who can access it. The information will only be used for the purpose of health and care research, or to contact you about future opportunities to participate in research. It will not be used to make decisions about future services available to you, such as insurance. If there is a risk that you could be identified, your data will only be used in research that has been independently reviewed by an ethics committee.

Your rights to access, change or move your information are limited, as we need to manage it in specific ways so that the research is reliable and accurate. If you withdraw from the study, we will keep the information about you that we already hold. To safeguard your rights, we will use the minimum personally identifiable information possible.

We use personally identifiable information to conduct research to improve health, care and services. As a publicly funded organisation, we have to ensure that it’s in the public interest when we use personally-identifiable information from people who have agreed to take part in research. We have to demonstrate that our research serves the interests of society as a whole. We do this by following the UK Policy Framework for Health and Social Care Research.

If you wish to complain about how we’ve handled your personal data, you can contact our Data Protection Officer who will investigate the matter. If you are not satisfied with our response or believe we are processing your personal data in a way that is not lawful, you can complain to the Information Commissioner’s Office.
