# Supplementary material for: Extended-release pharmacotherapy for opioid use disorder (EXPO): protocol for an open-label randomised controlled trial of the effectiveness and cost-effectiveness of injectable buprenorphine versus sublingual tablet buprenorphine and oral liquid methadone
Source: Trials. 2022 Aug 19;23:697. doi: 10.1186/s13063-022-06595-0 (PMC9389497; doi:10.1186/s13063-022-06595-0)
Supplement: Supplementary file 3 — Additional file 3. [file 13063_2022_6595_MOESM3_ESM.docx]

| **EX**tended-release **P**harmacotherapy for **O**pioid Use Disorder (**EXPO)** |
| --- |

**PARTICIPANT CONSENT FORM (VERSION 2.0 (08.01.20); REC: 19/LO/0483)**

**SITE NAME: South London and Maudsley NHS Trust**

**PLEASE WRITE YOUR INITIALS IN BLACK INK IN THE BOX TO ACCEPT**

| 1. I have read and understood the EXPO Participant Information Sheet (South London), dated   08^th^ January 2020 (version 2.0). I have had the opportunity to consider the information, ask  questions and have had these answered satisfactorily. Therefore, I agree to take part in the study.   1. I understand that taking part is voluntary and will not affect my treatment here. I understand that   I am free to withdraw from the study at any time without giving a reason. This decision does not affect  my rights to receive care and support services or my legal rights.   1. I agree to my GP (General Practitioner) being informed of my participation in the study. 2. I understand that if I receive additional counselling that sessions will be tape recorded to ensure   that these are being delivered correctly. I will be asked each time to give consent.   1. I understand that the data collected for the study will be published and summaries of the data   may be used by the manufacturer of extended-release buprenorphine so that this medication could  be made available and approved for use in the UK and elsewhere.   1. I understand that where it is relevant to EXPO, information will be taken from my addiction clinic   records by members of the EXPO research team and data collected during the study, may be  looked at by individuals from regulatory authorities, or the NHS Trust, or study sponsors.  I give permission for these individuals to do this.   1. I understand that the personal information collected about me for this trial will be stored securely   for 10 years or more at King's College London.   1. I consent to sharing my personal information with Payment Card Solutions (UK) Ltd for the registration   of my prepaid card for research payments. I understand there could be a delay of up to 1 week from  the research visit to when the funds are available on the prepaid card.   1. I consent to the sharing of my ID information with Public Health England so the researchers can   access data on drug treatment (NDTMS), NHS Hospital Episode Statistics, NHS Digital  (deaths register) and the Police National Computer. ***[OPTIONAL CONSENT- To reject mark X]*** |
| --- |

| PARTICIPANT:  **Name** [CAPS] ____________________________  **Signature** _______________________________ **Date** [DD/MM/YY] ______ / _____ / ______  PRINCIPAL/SUB INVESTIGATOR:    **Name** [CAPS]: ____________________________ **PARTICIPANT EXPO PIN**  **Signature** ________________________________ **Date** [DD/MM/YY] ______ / _____ / ______ |
| --- |

***COMPLETE 2 COPIES: 1 TO BE RETURNED TO THE PARTICIPANT; 1 TO BE FILED WITHIN THE EXPO INVESTIGATOR SITE FILE AND A COPY FILED IN THE PARTICIPANTS MEDICAL NOTES***
